# False equivalence: differences in the *in vitro* activity of ampicillin–sulbactam and amoxicillin–clavulanate in several Enterobacterales species

**DOI:** 10.1093/jacamr/dlaf147

**Published:** 2025-09-04

**Authors:** Sarah M Schrader, Carmela Bacani, Stanley Diaz, Yingzhe Kuang, Bulent Oral, Sarah Russell, Lars F Westblade, Michael J Satlin

**Affiliations:** Weill Cornell/Rockefeller/Sloan Kettering Tri-Institutional MD-PhD Program, New York, NY, USA; Department of Pathology, Brigham and Women’s Hospital, Boston, MA, USA; Department of Pathology, Massachusetts General Hospital, Boston, MA, USA; Laboratory Information Services, NewYork-Presbyterian Hospital, New York, NY, USA; Laboratory Information Services, NewYork-Presbyterian Hospital, New York, NY, USA; Laboratory Information Services, NewYork-Presbyterian Hospital, New York, NY, USA; Laboratory Information Services, NewYork-Presbyterian Hospital, New York, NY, USA; Laboratory Information Services, NewYork-Presbyterian Hospital, New York, NY, USA; Department of Medicine, Weill Cornell Medicine, New York, NY, USA; Department of Pathology and Laboratory Medicine, Weill Cornell Medicine, New York, NY, USA; Department of Pediatrics, Weill Cornell Medicine, New York, NY, USA; Department of Medicine, Weill Cornell Medicine, New York, NY, USA; Department of Pathology and Laboratory Medicine, Weill Cornell Medicine, New York, NY, USA

## Abstract

**Background and objectives:**

The β-lactam/β-lactamase inhibitor combinations ampicillin–sulbactam (SAM) and amoxicillin–clavulanate (AMC) are frequently used to treat Enterobacterales infections and are often assumed to be interchangeable, leading some clinical microbiology laboratories to report antimicrobial susceptibility testing (AST) results for only one of these agents. Given differences in β-lactamase inhibition between sulbactam and clavulanate, we hypothesized that the *in vitro* activities of SAM and AMC may differ.

**Methods:**

To understand the prevalence of discordant SAM and AMC susceptibility results in Enterobacterales species, we analysed AST results obtained by broth microdilution (MicroScan WalkAway, Beckman Coulter) for Enterobacterales isolates recovered from clinical specimens between 2018 and 2022 at an academic medical centre in New York City.

**Results:**

Percentages of isolates susceptible to SAM were lower than percentages susceptible to AMC for *Escherichia coli* (58.1% versus 85.4% of 23,746 isolates, *P* < 0.0001), *Klebsiella pneumoniae* group (76.7% versus 88.7% of 6,630, *P* < 0.0001), *Proteus mirabilis* (88.0% versus 95.4% of 3,185, *P* < 0.0001) and *Klebsiella oxytoca* (69.6% versus 90.7% of 890, *P* < 0.0001). Isolates of *E. coli*, *K. pneumoniae* group and *P. mirabilis* with susceptibility profiles consistent with ESBL production (ceftriaxone resistant and cefoxitin susceptible) were more likely to be susceptible to AMC but not susceptible to SAM than isolates without this susceptibility phenotype (*E*. *coli*, 38% versus 27%, *P* < 0.0001; *K. pneumoniae* group: 40% versus 10%, *P* < 0.0001; *P. mirabilis*: 24% versus 6%, *P* < 0.0001).

**Conclusions:**

The high prevalence of SAM-not susceptible, AMC-susceptible isolates supports reporting AST results for both SAM and AMC to maximize options for antimicrobial therapy and to support antimicrobial stewardship.

## Introduction

The order Enterobacterales is a diverse collection of enteric Gram-negative bacteria that harbours several genera of clinical importance, including *Citrobacter*, *Escherichia*, *Klebsiella* and *Proteus*, among others.^1^ Members of the Enterobacterales are among the most common causes of urinary tract, bloodstream, respiratory and gastrointestinal infections.^1^ Due to widespread intrinsic and acquired β-lactam resistance mediated by β-lactamases, β-lactam/β-lactamase inhibitor combinations (βL-βLIs) are frequently considered for the treatment of infections caused by Enterobacterales. The aminopenicillin β-lactam/first-generation β-lactamase inhibitor combinations ampicillin–sulbactam (SAM) and amoxicillin–clavulanate (AMC) are often chosen due to their wide availability, low cost and favourable toxicity and tolerability profiles. The Clinical Laboratory Standards Institute (CLSI) includes SAM and AMC among the antimicrobial agents recommended for primary testing and reporting in clinical microbiology laboratories when performing antimicrobial susceptibility testing (AST) on Enterobacterales isolates.^2^ In clinical practice, SAM and AMC are often considered to have comparable clinical efficacy, leading many clinical microbiology laboratories to routinely test susceptibility to and/or report susceptibility results for only one of these two agents. This practice is supported by the grouping of the two agents together in Table 1A (‘Antimicrobial Agents that Should Be Considered for Testing and Reporting by Microbiology Laboratories, Enterobacterales’) of the 2025 CLSI M100 Performance Standards for Antimicrobial Susceptibility Testing (35th ed).^2^ While this grouping does not denote interchangeability, it does suggest similar interpretive categories and clinical efficacy. Further, the manual notes that ‘a laboratory will often test only one agent from a box routinely’, although decisions regarding which agents to include in routine AST are at the discretion of individual laboratories.^2^

Despite the perceived equivalence of these agents, there are differences in the β-lactamase inhibitor properties of sulbactam and clavulanate.^3^ Moreover, several reports have demonstrated that SAM and AMC susceptibility results for clinical isolates of Enterobacterales members *Escherichia coli*^4–10^ and *Klebsiella* species^6^ are often discordant, with susceptibility to AMC more prevalent than to SAM. In addition, despite placing the two agents together in the same box, the CLSI M100 does not include an ‘or’ between their names, which would indicate sufficient cross-resistance and cross-susceptibility to use susceptibility results for one agent to predict those of the other.^2^ These observations suggest that considering SAM and AMC as interchangeable and routinely reporting susceptibility results for only one of the two agents could result in selection of an ineffective therapy if cross-susceptibility is assumed or exclusion of a potentially effective therapy if cross-resistance is assumed. Understanding which Enterobacterales species exhibit discordant SAM and AMC susceptibility, as well as the direction and prevalence of this discordance, is key to informing reporting practices to optimize antimicrobial therapy and support antimicrobial stewardship efforts.

To explore the extent of discordance in SAM and AMC susceptibility results among clinical Enterobacterales isolates, we analysed contemporary SAM and AMC susceptibility data for Enterobacterales isolates recovered in a large, urban clinical microbiology laboratory.

## Methods

### Data collection

We retrospectively queried the laboratory information system of NewYork-Presbyterian/Weill Cornell Medical Center (NYP/WCMC) for Enterobacterales isolates collected between 1 January 2018 and 31 December 2022 and corresponding susceptibility results for ampicillin (AMP), SAM and AMC. A five-year period was selected to ensure a large set of isolates encompassing a range of species. Laboratory sites comprised the main NYP/WCMC campus, an 862-bed quaternary care facility; NYP Lower Manhattan Hospital, a 180-bed community hospital; NYP Westchester Behavioral Health Center, a psychiatric facility; and a range of outpatient offices affiliated with NYP/WCMC. AST was performed using the Negative MIC Panel Type 42 on the MicroScan WalkAway system (Beckman Coulter, Brea, CA, USA).

### Isolate selection

We included isolates of Enterobacterales species without intrinsic resistance to either SAM or AMC,^2^ except for *Salmonella* and *Shigella*, for which SAM and AMC are not recommended for routine AST.^2^ Isolates were obtained from clinical specimens sent to the clinical microbiology laboratory for diagnostic purposes and identified to the species level by matrix-assisted laser desorption/ionization time-of-flight mass spectrometry (Bruker, Billerica, MA, USA). *Citrobacter amalonaticus*, *Citrobacter farmeri* and *Citrobacter sedlakii* were grouped together as *C. amalonaticus* complex, and *Klebsiella pneumoniae* and *Klebsiella variicola* were grouped together as *K. pneumoniae* group. Organism groups for which the total number of isolates across all five years analysed was less than 30 were excluded. Individual isolates missing MicroScan results for any of the three selected antibiotics were excluded. In addition, to reduce the chances of including duplicate organisms, if multiple isolates of the same species were recovered from the same patient in a single year, only one isolate was included. Isolates collected from patients without medical record numbers, missing information on the patient location at the time of collection and/or missing information on the source of the specimen from which the isolate was recovered were excluded.

### Interpretive categories

We determined interpretive susceptibility categories from the MicroScan MIC values using the MIC breakpoints for Enterobacterales listed in the 31st edition of the CLSI M100 Performance Standards for Antimicrobial Susceptibility Testing (Table 1),^11^ which reflect the breakpoints in use in the clinical laboratory at the time this study was initiated. The MIC breakpoints for AMP, SAM and AMC listed in the 31st edition of the M100 have remained unchanged in the 32nd–35th editions.^2,12–14^ Moreover, these CLSI breakpoints match the EUCAST susceptible breakpoints for the intravenous formulations of SAM and AMC.^15^ ‘Not susceptible’ in this manuscript refers to isolates that fall into either the intermediate or resistant interpretive category.

**Table 1.** Enterobacterales MIC breakpoints used in this study, from Table 2A of the 31st edition of the CLSI M100^11^

	Interpretive categories		
Antibiotic	Susceptible	Intermediate	Resistant
AMP	≤8 µg/mL	16 µg/mL	≥32 µg/mL
SAM	≤8/4 µg/mL	16/8 µg/mL	≥32/16 µg/mL
AMC	≤8/4 µg/mL	16/8 µg/mL	≥32/16 µg/mL

AMP, ampicillin; SAM, ampicillin–sulbactam; AMC, amoxicillin–clavulanate.

### Extended-spectrum β-lactamase-producer subgroup analysis

For the extended-spectrum β-lactamase (ESBL)-producer subgroup analysis, no formal ESBL testing results were available, so presumed ESBL-producing isolates were defined as those resistant to ceftriaxone and susceptible to cefoxitin as a proxy.^16^ Isolates for which ceftriaxone and/or cefoxitin susceptibility results were unavailable were excluded from this subgroup analysis.

### Statistical analysis

For comparisons of percent susceptibility to SAM versus percent susceptibility to AMC within each organism group, statistical significance was determined by the McNemar test with Yates’s continuity correction. For comparisons of percentages of isolates not susceptible to SAM and susceptible to AMC between ESBL-producing and non-ESBL-producing organisms, statistical significance was determined by Fisher’s exact test in GraphPad Prism 10 (Dotmatics, Boston, MA, USA). *P* values were adjusted for multiple comparisons using the Bonferroni correction. Linear regressions were performed in GraphPad Prism 10.

## Results

### SAM and AMC susceptibility rates for clinically important Enterobacterales

**Figure 1.**
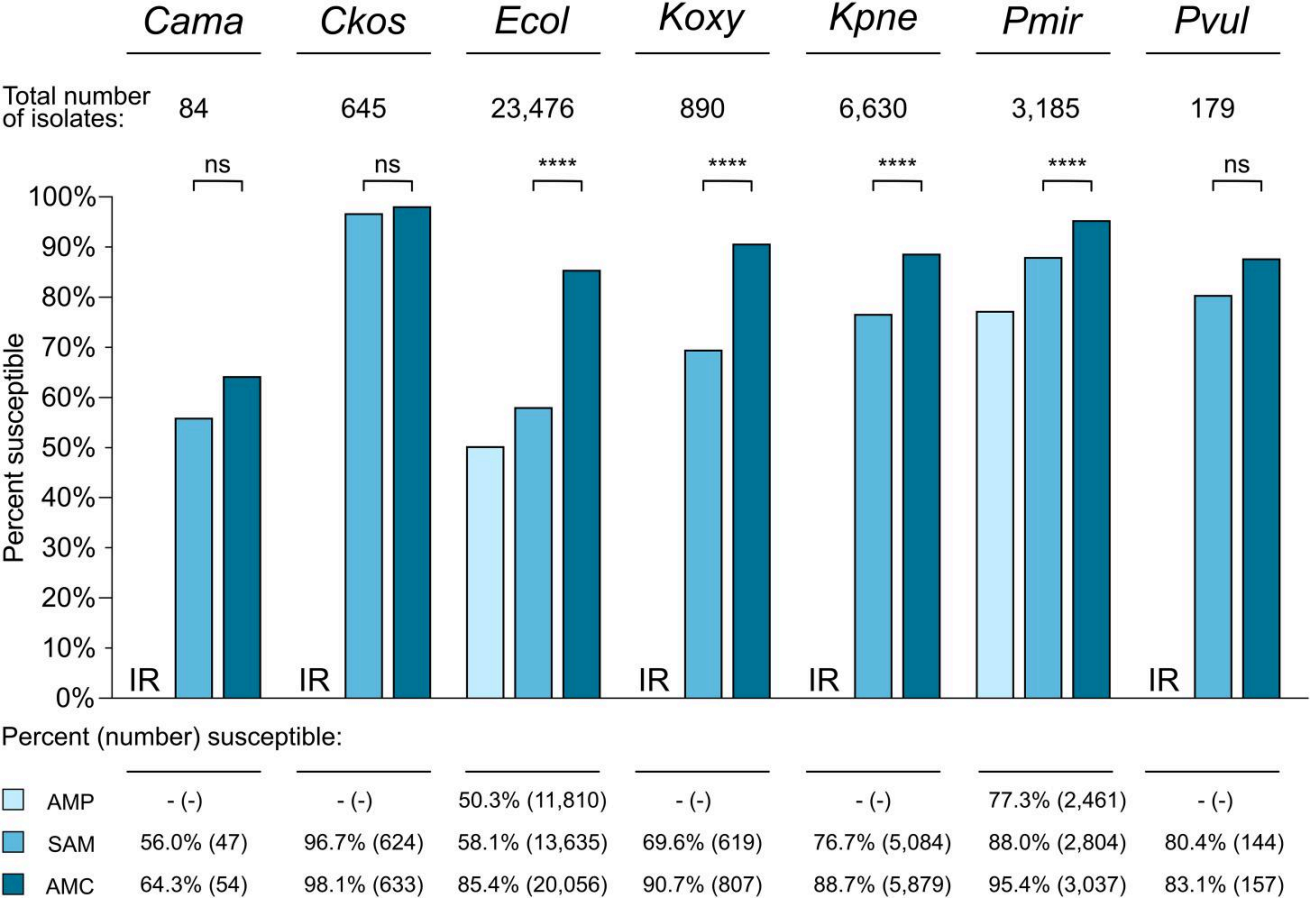
Susceptibility of Enterobacterales clinical isolates recovered from 2018–22 to ampicillin, amoxicillin–clavulanate and ampicillin–sulbactam. *Cama*, *Citrobacter amalonaticus* complex; *Ckos*, *Citrobacter koseri*; *Ecol*, *Escherichia coli*; *Koxy*, *Klebsiella oxytoca*; *Kpne*, *Klebsiella pneumoniae* group; *Pmir*, *Proteus mirabilis*; *Pvul*, *Proteus vulgaris*; AMP, ampicillin (excluded for organisms that are considered intrinsically resistant, IR); AMC, amoxicillin–clavulanate; SAM, ampicillin–sulbactam. Bars represent percent of isolates susceptible to the antibiotic. Percentages and absolute numbers of susceptible isolates are listed below the graph. The total number of isolates for each organism group is listed at the top of the graph below the organism group name. Significance of differences between percent susceptibility to SAM and AMC was determined by the McNemar test with Yates’s continuity correction. *P* values were adjusted for multiple comparisons using the Bonferroni correction. ****, *P* < 0.0001; ns, not significant (*P *> 0.05).

The following species/organism categories met our inclusion criteria: *C. amalonaticus* complex (84 isolates); *Citrobacter koseri* (645 isolates); *E. coli* (23,476 isolates); *Klebsiella oxytoca* (890 isolates); *K. pneumoniae* group (6,630 isolates); *Proteus mirabilis* (3,185 isolates); and *Proteus vulgaris* (179 isolates). We first compared the percentage of isolates susceptible to AMP, AMC and SAM for each organism group (Figure 1). We found that SAM susceptibility rates were significantly lower than AMC susceptibility rates for *E. coli* (58.1% versus 85.4%, *P* < 0.0001), *K. oxytoca* (69.6% versus 90.7%, *P* < 0.0001), *K. pneumoniae* group (76.7% versus 88.7%, *P* < 0.0001) and *P. mirabilis* (88.0% versus 95.4%, *P* < 0.0001). Of these four organism groups, we observed the largest absolute difference (27.3%) for *E. coli* and the smallest difference (7.4%) for *P. mirabilis*.

### Distribution of SAM and AMC susceptibility profiles

**Figure 2.**
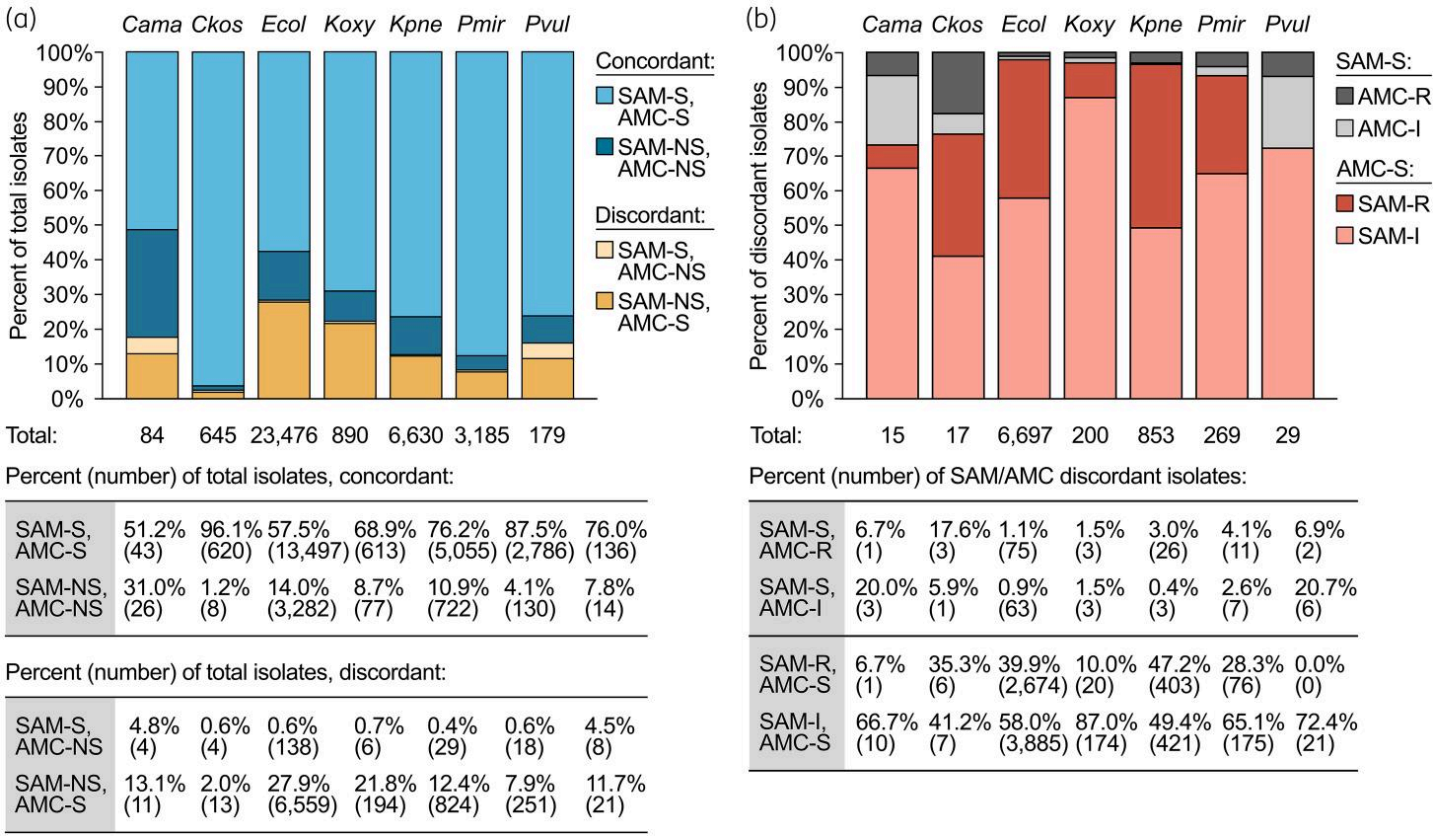
Discordance in ampicillin–sulbactam and amoxicillin–clavulanate susceptibility categories for Enterobacterales clinical isolates. (a) Distribution of ampicillin–sulbactam (SAM) and amoxicillin–clavulanate (AMC) susceptibility profiles among Enterobacterales: *Cama*, *Citrobacter amalonaticus* complex; *Ckos*, *Citrobacter koseri*; *Ecol*, *Escherichia coli*; *Koxy*, *Klebsiella oxytoca*; *Kpne*, *Klebsiella pneumoniae* group; *Pmir*, *Proteus mirabilis*; *Pvul*, *Proteus vulgaris*. The two concordant categories represent isolates that have the same susceptibility classification (susceptible, S, or not susceptible, NS) for both SAM and AMC. The two discordant categories represent isolates that are susceptible to one antibiotic and not susceptible to the other. The total number of isolates in each organism group is listed below each bar. Percentages and absolute numbers of isolates with each susceptibility profile are listed below the graph. (b) Breakdown of the not susceptible component into resistant (R) and intermediate (I) subcategories for the isolates with discordant SAM and AMC results. Percentages and absolute isolate numbers in each category are listed below the graph and in Tables S1–S7.

Our initial analysis, which considered aggregate susceptibility percentages among all isolates in each organism group, suggested that isolates not susceptible to SAM but susceptible to AMC comprise a substantial proportion of total clinical isolates for these four organism groups. To examine the SAM and AMC susceptibility profiles of individual isolates, we categorized each isolate by SAM and AMC interpretive categories (Figure 2, Tables S1–S7 (available as Supplementary data at *JAC-AMR* Online) and defined discordant categories as those in which an isolate was susceptible to one agent but not susceptible (*i.e.*, intermediate or resistant) to the other. In accordance with the lower percentages of isolates susceptible to SAM compared to AMC in the aggregate analysis, almost all isolates with discordant SAM and AMC susceptibility categories were SAM not susceptible and AMC susceptible (Figure 2a). This category comprised 27.9% of *E. coli* isolates, 21.8% of *K. oxytoca* isolates, 12.4% of *K. pneumoniae* group isolates and 7.9% of *P. mirabilis* isolates and represented the most common direction of discordance across all seven organism groups. Among SAM-not susceptible and AMC-susceptible isolates, the most common profile was SAM intermediate and AMC susceptible for all organism groups (Figure 2b), but SAM-resistant and AMC-susceptible isolates were also present and were especially common among *E. coli* (11.4% of total isolates, 39.9% of discordant isolates) and *K. pneumoniae* group (6.1% of total isolates, 47.2% of discordant isolates). Although absolute numbers were small, all organism groups also contained isolates that were SAM susceptible and AMC not susceptible. These isolates were proportionally most common among *C. amalonaticus* group (4.8% of total isolates) and *P. vulgaris* (4.5% of total isolates).

### Temporal trends in SAM and AMC susceptibility rates among Enterobacterales clinical isolates

**Figure 3.**
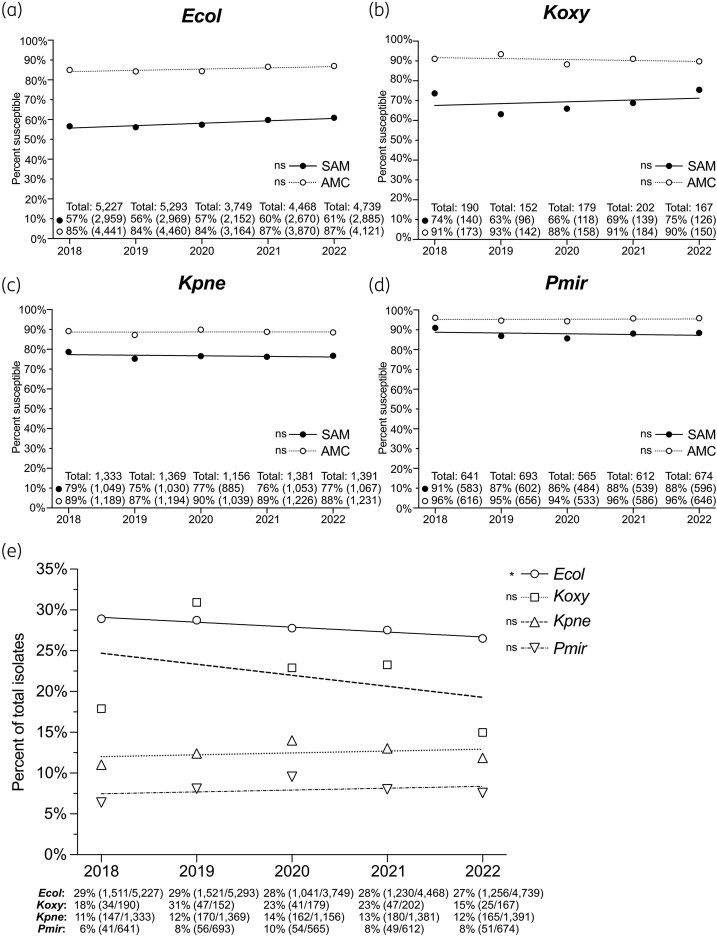
Susceptibility of Enterobacterales isolates to ampicillin–sulbactam and amoxicillin–clavulanate by year (2018–22). (a–d) Percent of isolates susceptible to ampicillin–sulbactam (SAM, solid lines, black circles) and amoxicillin–clavulanate (AMC, dotted lines, white circles) for (a) *Escherichia coli* (*Ecol*), (b) *Klebsiella oxytoca* (*Koxy*), (c) *Klebsiella pneumoniae* group (*Kpne*) and (d) *Proteus mirabilis* (*Pmir*). Circles mark the percentage of isolates from each year susceptible to the antibiotic. For each year, the total number of isolates and percentage of susceptible isolates followed by absolute number of susceptible isolates in parentheses is shown at the bottom of the graph above the *x*-axis. Linear regression lines for each antibiotic are shown. Statistical significance of the slope of each regression line is indicated to the left of the corresponding line and symbol in the legend at the lower right of each graph. *P* values were adjusted for multiple comparisons using the Bonferroni correction. ns, slope not significantly different from zero (*P*>0.05). (e) Percent of isolates not susceptible to SAM and susceptible to AMC per year by organism group. Symbols (*Ecol*, open circles; *Koxy*, open squares; *Kpne*, open upward triangles; *Pmir*, open downward triangles) represent percent of total isolates. For each organism group, percentages are printed below the *x*-axis followed by the absolute number of isolates in the SAM-not susceptible, AMC-susceptible category over the total number of isolates in parentheses. The linear regression line for each organism group is shown. Statistical significance of the slope of each regression line is indicated to the left of the corresponding line and symbol in the legend to the right of the graph. *P* values were adjusted for multiple comparisons using the Bonferroni correction. *, slope deviates from zero with a *P* value of <0.05; ns, slope not significantly different from zero (*P*>0.05).

To visualize temporal trends, we plotted the percentage of isolates susceptible to SAM and AMC by year (2018–22) for each of the four organism groups with a statistically significant difference in SAM versus AMC susceptibility (Figure 3a–d). Percentages of isolates susceptible to SAM were less than corresponding percentages of isolates susceptible to AMC across all years for all four organism groups, and linear regression analysis showed no significant trends (Figure 3a–d).

We also plotted the percentage of isolates in each of the four organism groups that were SAM-not susceptible, AMC-susceptible for each year (Figure 3e). *Escherichia coli*, *K. pneumoniae* group and *P. mirabilis* exhibited low year-to-year variability in the percentage of SAM-not susceptible, AMC-susceptible isolates. Percentages for *K. oxytoca* were more variable, likely because absolute numbers were smallest for this organism group. Linear regression analysis showed no significant trends except for a small negative trend (slope −0.006015 ± 0.002604) for *E. coli* over time. Together, these results demonstrate that the prevalence of SAM-not susceptible, AMC-susceptible Enterobacterales isolates is stable from year to year and not attributable to a transient anomaly.

### SAM and AMC susceptibility rates in isolates from different clinical settings

To understand whether clinical setting influences the prevalence of discordant SAM and AMC susceptibilities, we separated the isolates in each organism group by the location of the patient upon collection of the specimen from which the isolate was recovered [outpatient, emergency, non-intensive care unit (ICU) inpatient and ICU] (Table S8) or the source from which the isolate was recovered (blood, respiratory, urine, wound/tissue/body fluid and other) (Table S9). For the four organism groups with a statistically significant difference in SAM versus AMC susceptibility rates (Figure 1), a statistically significant difference was maintained across all patient location subgroups. For *E. coli* and *K. oxytoca*, a statistically significant difference in SAM versus AMC susceptibility rates was also observed across all sources for which the conditions for statistical testing were met. *Klebsiella pneumoniae* group exhibited a statistically significant difference for all source categories except ‘other’, while *P. mirabilis* exhibited a significant difference for only urine and wound/tissue/body fluid source categories. Overall, the source sub-analysis suggests that SAM-not susceptible, AMC-susceptible organisms are not restricted to a particular body site and are similarly prevalent in outpatients as well as inpatients admitted to both hospital wards and ICUs.

### Rates of SAM non-susceptibility and AMC susceptibility in presumed ESBL-producing Enterobacterales isolates

**Figure 4.**
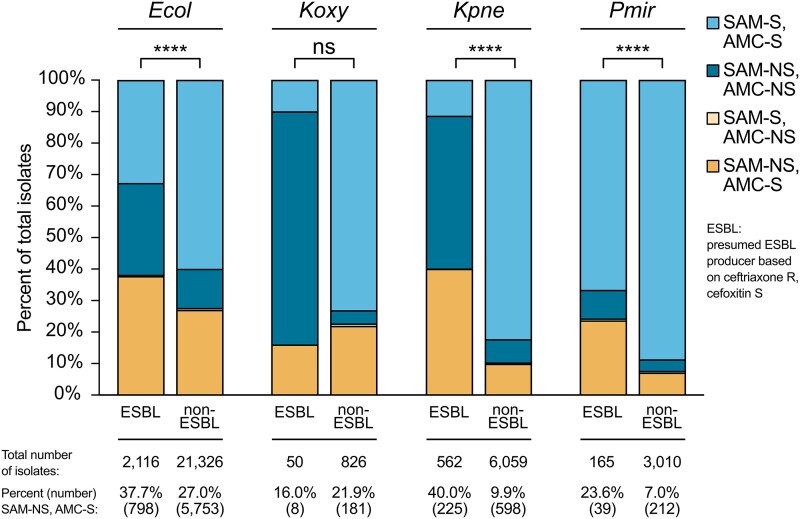
Ampicillin–sulbactam and amoxicillin–clavulanate susceptibility profiles for Enterobacterales clinical isolates by inferred extended-spectrum β-lactamase (ESBL) status. Presumed ESBL producers were defined as isolates resistant (R) to ceftriaxone and susceptible (S) to cefoxitin. Isolates for which susceptibility results for ceftriaxone and/or cefoxitin were not available were excluded. Bars show the distribution of ampicillin–sulbactam (SAM) and amoxicillin–clavulanate (AMC) susceptibility profiles for presumed ESBL producers (ESBL) and presumed non-ESBL producers (non-ESBL) for *Escherichia coli* (*Ecol*), *Klebsiella oxytoca* (*Koxy*), *Klebsiella pneumoniae* group (*Kpne*) and *Proteus mirabilis* (*Pmir*). Percent of isolates that were SAM not susceptible (NS) and AMC susceptible (S) was compared between ESBL and non-ESBL isolates in each organism group using Fisher’s exact test. ****, *P* < 0.0001; ns, not significant (*P*>0.05). Total number of isolates in each category (ESBL or non-ESBL) along with percentages and absolute numbers of isolates with the SAM-NS, AMC-S profile are listed below the graph.

We next hypothesized that ESBL production was associated with the SAM-not susceptible, AMC-susceptible phenotype. To explore this, we identified presumed ESBL-producing isolates in the four organism groups with a statistically significant difference in SAM versus AMC susceptibility rates based on ceftriaxone resistance with cefoxitin susceptibility.^16^ We then compared the percentage of isolates that were SAM-not susceptible and AMC-susceptible between the presumed ESBL-producing isolates and non-ESBL-producing isolates for each organism group (Figure 4). The ESBL subgroups were significantly enriched for SAM-not susceptible and AMC-susceptible isolates for *E. coli* (37.7% versus 27.0%; *P* < 0.0001), *K. pneumoniae* group (40.0% versus 9.9%; *P* < 0.0001) and *P. mirabilis* (18.4% versus 5.5%; *P* < 0.0001) but not for *K. oxytoca* (16.0% versus 21.9%, *P*>0.05). Enrichment of SAM-not susceptible, AMC-susceptible isolates among presumed ESBL producers suggests that ESBL production is associated with this phenotype in *E. coli*, *K. pneumoniae* group and *P. mirabilis*. The clear differences in the SAM and AMC susceptibility profiles among presumed ESBL-producing isolates of each organism group likely reflect differences in the types of ESBLs carried by members of each organism group. For example, most presumed ESBL-producing *K. oxytoca* isolates are not susceptible to both SAM and AMC, while most ESBL-producing *P. mirabilis* isolates remain susceptible to both SAM and AMC.

## Discussion

Our analysis of Enterobacterales isolates collected in multiple clinical settings over five years provides evidence that SAM-not susceptible, AMC-susceptible organisms comprise a substantial percentage—ranging from 7.4% to 27.9%—of *E. coli*, *K. oxytoca*, *K. pneumoniae* group and *P. mirabilis* clinical isolates. Previous reports of SAM-not susceptible, AMC-susceptible clinical Enterobacterales isolates have mostly focused on *E. coli* and have had relatively small sample sizes.^4–10^ Of these studies, the largest analysed 823 *E. coli* isolates and 150 *Klebsiella* species isolates collected over a six-month period and found that 11% of both *E. coli* and *Klebsiella* species isolates were SAM not susceptible, AMC susceptible.^6^ Our analysis builds upon these prior reports by including a larger number of *E. coli* clinical isolates as well as clinical isolates of *K. oxytoca*, *K. pneumoniae* group and *P. mirabilis.* We also show that the prevalence of SAM-not susceptible, AMC-susceptible isolates has remained relatively constant over a five-year period and does not depend on clinical location or source material, suggesting that these isolates are ubiquitous across clinical settings and that their prevalence among isolates included in our study is unlikely to be attributable to a transient phenomenon.

The substantial prevalence of SAM-not susceptible, AMC-susceptible isolates of *E. coli*, *K. oxytoca*, *K. pneumoniae* group and *P. mirabilis* reported here suggests that SAM and AMC should not be considered interchangeable when reporting susceptibility data for these organisms. Moreover, considering SAM and AMC interchangeable could have heretofore unrecognized clinical consequences. If only results for AMC are reported, a susceptible result might offer false reassurance that the isolate is also susceptible to SAM, meaning that patients who require an intravenous antibiotic option could receive ineffective therapy if SAM is used. If only results for SAM are reported, AMC might be excluded from consideration for organisms that are susceptible to AMC, thus unnecessarily eliminating a relatively inexpensive, orally available therapeutic option for infections due to these organisms. Although oral AMC (the only formulation available in the USA) may not reliably achieve pharmacokinetic–pharmacodynamic (PK-PD) targets associated with bactericidal activity in systemic infections due to Enterobacterales, oral AMC represents an important treatment option for uncomplicated urinary tract infections due to these organisms and to complete therapy for systemic infections.^17^ Moreover, intravenous AMC, which is available in many countries outside of the USA, provides greater PK exposures than the oral formulation and is an option for primary treatment of systemic infections due to Enterobacterales.^17^

Our results have practical implications for clinical microbiology laboratories. Many laboratories rely on commercial automated AST systems such as the MicroScan Walkaway system, the BD Phoenix system (Becton Dickinson, Franklin Lake, NJ, USA) and the VITEK 2 system (bioMérieux, Marcy-l’Étoile, France). All three systems offer multiple panels designed for AST of Gram-negative bacteria, including Enterobacterales. Some panels include only AMC or SAM, while others include both. Laboratories that implement automated AST systems must decide which panels to use and which results to release for each group of organisms. Our findings argue in favour of selecting panels that test for both AMC and SAM. Laboratories that select a panel with only one agent should consider including a comment to highlight the non-equivalence of the two agents when reporting results and making an alternative test, such as disk diffusion, available for the other agent upon clinician request. If panels that include both agents are used, our findings suggest that results for both agents should be released to clinicians instead of suppressing one of the results.

The predominant mechanism of resistance to β-lactams among Enterobacterales is via production of β-lactamases, which enzymatically inactivate β-lactams. β-lactamases carried by Enterobacterales vary widely in structure, molecular mechanism, substrate specificity and susceptibility to β-lactamase inhibitors,^16,18^ resulting in a variety of possible resistance patterns. Although mechanistic exploration is beyond the scope of the current study, the prevalence of SAM-not susceptible, AMC-susceptible isolates among Enterobacterales supports prior work that found that clavulanate is often more effective at inhibiting Enterobacterales-borne β-lactamases than sulbactam.^3,5^ For example, the concentration of clavulanate required to inhibit TEM-1 and SHV-1 β-lactamases (the most common β-lactamases in *E. coli* and *K. pneumoniae*, respectively) is 50–500 times lower than the concentration of sulbactam required to inhibit the same enzymes.^3^ Moreover, our finding that presumed ESBL producers are enriched for SAM-not susceptible, AMC-susceptible isolates compared to non-ESBL producers is consistent with prior work demonstrating that clavulanate is also a more potent inhibitor of ESBL TEM and SHV variants than sulbactam and that clavulanate lowers ampicillin MIC values more than sulbactam in ESBL-producing *E. coli*:^3,19^ Clavulanate and sulbactam have also shown different properties against CTX-M enzymes, which are the most common ESBLs worldwide and in the Middle Atlantic region of the USA where the study was conducted.^20^ For example, while clavulanate is an effective inhibitor of CTX-M-14, sulbactam is a poor inhibitor and is actually hydrolyzed by this enzyme.^21^ To further elucidate mechanisms underlying the SAM-not susceptible, AMC-susceptible resistance pattern, future studies could focus on defining the β-lactamase profiles associated with SAM-not susceptible, AMC-susceptible isolates.

Our study has several limitations. First, SAM and AMC susceptibility results were determined using a single automated method, MicroScan, and not by a reference AST method. However, a recent study that compared results of different AST methods for 200 Enterobacterales isolates found that the MicroScan Neg MIC 56 Panel had an essential agreement of >95% compared to reference broth microdilution for SAM and AMC, suggesting that similar results would likely have been observed using reference broth microdilution.^22^ Second, our analysis included relatively low numbers of *C. amalonaticus* complex and *P. vulgaris* isolates, which may have precluded detection of a significant difference in rates of susceptibility to SAM and AMC. Third, ESBL-producer status in our ESBL sub-analysis was based on a proxy (ceftriaxone resistance along with cefoxitin susceptibility) rather than the results of formal ESBL testing. Some isolates may have been resistant to ceftriaxone due to AmpC β-lactamases or hyperproduction of OXY β-lactamases in *K. oxytoca*.^20,23^ Fourth, although our analysis included isolates from a range of clinical settings, isolates were primarily from a single centre in New York City; additional studies at centres in other geographic locations will be required to establish the generalizability of these results. Finally, investigation of clinical outcomes associated with isolates with different SAM and AMC resistance profiles was outside the scope of our study and therefore not performed.

Despite these limitations, we believe that our results provide evidence in favour of reporting both SAM and AMC susceptibility testing results for Enterobacterales isolates that are not intrinsically resistant to these agents. If only one agent is tested, a comment noting non-equivalence should be added to discourage clinicians from making assumptions about the other agent. This is especially important for isolates that test SAM resistant, as our results suggest that AMC might remain a viable option for those isolates, or that test AMC susceptible, as our results suggest that SAM might not be a viable option for those isolates. These reporting practices will help optimize antibiotic therapy for individual patients and promote antibiotic stewardship.

## Supplementary Material

dlaf147_Supplementary_Data

## Transparency declarations

M.J.S. reports participation on a Data and Safety Monitoring Board for AbbVie and research funding through his institution from Merck, SNIPRBiome, bioMéreiux and Melinta Therapeutics. L.F.W. has received research funding from Accelerate Diagnostics, bioMérieux, Element Materials Technology, Hardy Diagnostics, Melinta Therapeutics, Roche Molecular Systems, Selux Diagnostics and SNIPRBiome; and served on advisory boards for Roche Molecular Systems, Shionogi and Talis Biomedical. All other authors: nothing to declare.

## Supplementary data

Supplementary Tables S1–S9 are available as Supplementary data at *JAC-AMR* Online.
