# Supplementary material for: False equivalence: differences in the in vitro activity of ampicillin–sulbactam and amoxicillin–clavulanate in several Enterobacterales species
Source: JAC Antimicrob Resist. 2025 Sep 4;7(5):dlaf147. doi: 10.1093/jacamr/dlaf147 (PMC12409269; doi:10.1093/jacamr/dlaf147)
Supplement: dlaf147_Supplementary_Data [file dlaf147_supplementary_data.docx]

**SUPPLEMENTARY MATERIALS**

**MANUSCRIPT TITLE: False equivalence: Differences in the in vitro activity of ampicillin-sulbactam and amoxicillin-clavulanate in several Enterobacterales species**

**AUTHORS:**

Sarah M. Schrader, Carmela Bacani, Stanley Diaz, Yingzhe Kuang, Bulent Oral, Sarah Russell, Lars F. Westblade, Michael J. Satlin

**CONTENTS:**

- Supplementary Tables 1-9

**Supplementary Table 1: Ampicillin-sulbactam and amoxicillin-clavulanate susceptibility categories for *Citrobacter amalonaticus* complex.** S, susceptible. I, intermediate. R, resistant. Percentages of isolates falling into each category are listed along with absolute numbers of isolates over total numbers of isolates in parentheses. The gray background represents categories for which ampicillin-sulbactam (SAM) and amoxicillin-clavulanate (AMC) susceptibility categories are discordant, *i.e.,* isolates are susceptible to one but not susceptible (*i.e.,* intermediate or resistant) to the other.

|  | | **AMC** | | |  |
| --- | --- | --- | --- | --- | --- |
|  |  | **S** | **I** | **R** | **Total** |
| **SAM** | **S** | 51.2%  (43/84) | 3.6%  (3/84) | 1.2%  (1/84) | 56.0%  (47/84) |
|  | **I** | 11.9%  (10/84) | 6.0%  (5/84) | 1.2%  (1/84) | 19.1%  (16/84) |
|  | **R** | 1.2%  (1/84) | 10.7%  (9/84) | 13.1%  (11/84) | 25.0%  (21/84) |
| **Total** | | 64.3%  (54/84) | 20.2%  (17/84) | 15.6%  (13/84) |  |

**Supplementary Table 2: Ampicillin-sulbactam and amoxicillin-clavulanate susceptibility categories for *Citrobacter koseri*.** S, susceptible. I, intermediate. R, resistant. Percentages of isolates falling into each category are listed along with absolute numbers of isolates over total numbers of isolates in parentheses. The gray background represents categories for which ampicillin-sulbactam (SAM) and amoxicillin-clavulanate (AMC) susceptibility categories are discordant, *i.e.,* isolates are susceptible to one but not susceptible (*i.e.,* intermediate or resistant) to the other.

|  | | **AMC** | | |  |
| --- | --- | --- | --- | --- | --- |
|  |  | **S** | **I** | **R** | **Total** |
| **SAM** | **S** | 96.1%  (620/645) | 0.2%  (1/645) | 0.5%  (3/645) | 96.7%  (624/645) |
|  | **I** | 1.1%  (7/645) | 0%  (0/645) | 0%  (0/645) | 1.1%  (7/645) |
|  | **R** | 0.9%  (6/645) | 0.5%  (3/645) | 0.8%  (5/645) | 2.2%  (14/645) |
| **Total** | | 98.1%  (633/645) | 0.6%  (4/645) | 1.2%  (8/645) |  |

**Supplementary Table 3: Ampicillin-sulbactam and amoxicillin-clavulanate susceptibility categories for *Escherichia coli*.** S, susceptible. I, intermediate. R, resistant. Percentages of isolates falling into each category are listed along with absolute numbers of isolates over total numbers of isolates in parentheses. The gray background represents categories for which ampicillin-sulbactam (SAM) and amoxicillin-clavulanate (AMC) susceptibility categories are discordant, *i.e.,* isolates are susceptible to one but not susceptible (*i.e.,* intermediate or resistant) to the other.

|  | | **AMC** | | |  |
| --- | --- | --- | --- | --- | --- |
|  |  | **S** | **I** | **R** | **Total** |
| **SAM** | **S** | 57.5%  (13,497/23,476) | 0.3%  (63/23,476) | 0.3%  (75/23,476) | 58.1%  (13,635/23,476) |
|  | **I** | 16.6%  (3,885/23,476) | 1.8%  (412/23,476) | 1.4%  (330/23,476) | 19.7%  (4,627/23,476) |
|  | **R** | 11.4%  (2,674/23,476) | 7.6%  (1,780/23,476) | 3.2%  (760/23,476) | 22.2%  (5,214/23,476) |
| **Total** | | 85.4%  (20,056/23,476) | 9.6%  (2,255/23,476) | 5.0%  (1,165/23,476) |  |

**Supplementary Table 4: Ampicillin-sulbactam and amoxicillin-clavulanate susceptibility categories for *Klebsiella oxytoca.*** S, susceptible. I, intermediate. R, resistant. Percentages of isolates falling into each category are listed along with absolute numbers of isolates over total numbers of isolates in parentheses. The gray background represents categories for which ampicillin-sulbactam (SAM) and amoxicillin-clavulanate (AMC) susceptibility categories are discordant, *i.e.,* isolates are susceptible to one but not susceptible (*i.e.,* intermediate or resistant) to the other.

|  | | **AMC** | | |  |
| --- | --- | --- | --- | --- | --- |
|  |  | **S** | **I** | **R** | **Total** |
| **SAM** | **S** | 68.9%  (613/890) | 0.3%  (3/890) | 0.3%  (3/890) | 69.6%  (619/890) |
|  | **I** | 20.0%  (174/890) | 0.1%  (1/890) | 0.3%  (3/890) | 20.0%  (178/890) |
|  | **R** | 2.3%  (20/890) | 5.0%  (44/890) | 3.3%  (29/890) | 10.4%  (93/890) |
| **Total** | | 90.7%  (807/890) | 5.4%  (48/890) | 3.9%  (35/890) |  |

**Supplementary Table 5: Ampicillin-sulbactam and amoxicillin-clavulanate susceptibility categories for *Klebsiella pneumoniae* group.** S, susceptible. I, intermediate. R, resistant. Percentages of isolates falling into each category are listed along with absolute numbers of isolates over total numbers of isolates in parentheses. The gray background represents categories for which ampicillin-sulbactam (SAM) and amoxicillin-clavulanate (AMC) susceptibility categories are discordant, *i.e.,* isolates are susceptible to one but not susceptible (*i.e.,* intermediate or resistant) to the other.

|  | | **AMC** | | |  |
| --- | --- | --- | --- | --- | --- |
|  |  | **S** | **I** | **R** | **Total** |
| **SAM** | **S** | 76.2%  (5,055/6,630) | 0.1%  (3/6,630) | 0.4%  (26/6,630) | 76.7%  (5,084/6,630) |
|  | **I** | 6.4%  (421/6,630) | 0.3%  (19/6,630) | 0.3%  (13/6,630) | 6.8%  (453/6,630) |
|  | **R** | 6.1%  (403/6,630) | 5.6%  (371/6,630) | 4.8%  (319/6,630) | 16.5%  (1,093/6,630) |
| **Total** | | 88.7%  (5,879/6,630) | 5.9%  (393/6,630) | 5.4%  (358/6,630) |  |

**Supplementary Table 6: Ampicillin-sulbactam and amoxicillin-clavulanate susceptibility categories for *Proteus mirabilis*.** S, susceptible. I, intermediate. R, resistant. Percentages of isolates falling into each category are listed along with absolute numbers of isolates over total numbers of isolates in parentheses. The gray background represents categories for which ampicillin-sulbactam (SAM) and amoxicillin-clavulanate (AMC) susceptibility categories are discordant, *i.e.,* isolates are susceptible to one but not susceptible (*i.e.,* intermediate or resistant) to the other.

|  | | **AMC** | | |  |
| --- | --- | --- | --- | --- | --- |
|  |  | **S** | **I** | **R** | **Total** |
| **SAM** | **S** | 87.5%  (2,786/3,185) | 0.2%  (7/3,185) | 0.4%  (11/3,185) | 88.0%  (2,804/3,185) |
|  | **I** | 5.5%  (175/3,185) | 0.4%  (13/3,185) | 1.3%  (42/3,185) | 7.2%  (230/3,185) |
|  | **R** | 2.4%  (76/3,185) | 1.7%  (53/3,185) | 0.7%  (22/3,185) | 4.7%  (151/3,185) |
| **Total** | | 95.4%  (3,037/3,185) | 2.3%  (73/3,185) | 2.4%  (75/3,185) |  |

**Supplementary Table 7: Ampicillin-sulbactam and amoxicillin-clavulanate susceptibility categories for *Proteus vulgaris*.** S, susceptible. I, intermediate. R, resistant. Percentages of isolates falling into each category are listed along with absolute numbers of isolates over total numbers of isolates in parentheses. The gray background represents categories for which ampicillin-sulbactam (SAM) and amoxicillin-clavulanate (AMC) susceptibility categories are discordant, *i.e.,* isolates are susceptible to one but not susceptible (*i.e.,* intermediate or resistant) to the other.

|  | | **AMC** | | |  |
| --- | --- | --- | --- | --- | --- |
|  |  | **S** | **I** | **R** | **Total** |
| **SAM** | **S** | 76.0%  (136/179) | 3.4%  (6/179) | 1.1%  (2/179) | 80.4%  (144/179) |
|  | **I** | 11.7%  (21/179) | 3.9%  (7/179) | 1.1%  (2/179) | 16.8%  (30/179) |
|  | **R** | 0%  (0/179) | 1.1%  (2/179) | 1.7%  (3/179) | 2.8%  (5/179) |
| **Total** | | 87.7%  (157/179) | 8.4%  (15/179) | 3.9%  (7/179) |  |

**Supplementary Table 8: Ampicillin-sulbactam and amoxicillin-clavulanate susceptibility rates by patient location.**

Percentages of isolates susceptible to ampicillin-sulbactam (SAM-S) or amoxicillin-clavulanate (AMC-S) are listed along with absolute numbers of isolates over total numbers of isolates in parentheses. Significance of differences between percent susceptibility to SAM and AMC was determined by the McNemar test with Yates’s continuity correction. P-values were adjusted for multiple comparisons using the Bonferroni correction. For each comparison, significance is indicated after the AMC susceptibility percentage: **, p < 0.01; ***, p < 0.001; superscript ns, not significant (p > 0.05); superscript N/A, McNemar test invalid because there were no isolates with discordant SAM and AMC results. ICU, intensive care unit.

|  |  | Location | | | |
| --- | --- | --- | --- | --- | --- |
|  |  | Outpatient | Emergency | Inpatient, non-ICU | Inpatient, ICU |
| *Citrobacter amalonaticus*  complex | SAM-S | 63%  (19/30) | 61%  (14/23) | 38%  (9/24) | 71%  (5/7) |
|  | AMC-S | 77%^ns^  (23/30) | 70%^ns^  (16/23) | 42%^ns^  (10/24) | 71%^N/A^  (5/7) |
| *Citrobacter*  *koseri* | SAM-S | 96%  (355/369) | 98%  (138/141) | 96%  (73/76) | 98%  (58/59) |
|  | AMC-S | 98%^ns^  (361/369) | 100%^ns^  (141/141) | 97%^ns^  (74/76) | 97%^ns^  (57/59) |
| *Escherichia*  *coli* | SAM-S | 60%  (8,049/13,405) | 57%  (3,721/6,551) | 54%  (1,502/2,782) | 49%  (363/738) |
|  | AMC-S | 87%***  (11,677/13,405) | 85%***  (5,546/6,551) | 81%***  (2,252/2,782) | 79%***  (581/738) |
| *Klebsiella oxytoca* | SAM-S | 73%  (293/403) | 69%  (130/188) | 66%  (113/172) | 65%  (83/127) |
|  | AMC-S | 95%***  (381/403) | 91%***  (171/188) | 86%***  (148/172) | 84%***  (107/127) |
| *Klebsiella pneumoniae*  group | SAM-S | 81%  (2,334/2,882) | 77%  (1,382/1,802) | 71%  (909/1,272) | 68%  (459/674) |
|  | AMC-S | 92%***  (2,661/2,882) | 89%***  (1,600/1,802) | 83%***  (1,061/1,272) | 83%***  (557/674) |
| *Proteus*  *mirabilis* | SAM-S | 90%  (1,351/1,500) | 87%  (796/915) | 86%  (494/576) | 84%  (163/194) |
|  | AMC-S | 97%***  (1,449/1,500) | 94%***  (863/915) | 94%***  (543/576) | 94%**  (182/194) |
| *Proteus*  *vulgaris* | SAM-S | 82%  (56/68) | 92%  (57/62) | 66%  (23/35) | 57%  (8/14) |
|  | AMC-S | 85%^ns^  (58/68) | 94%^ns^  (58/62) | 83%^ns^  (29/35) | 86%^ns^  (12/14) |

**Supplementary Table 9: Ampicillin-sulbactam and amoxicillin-clavulanate susceptibility rates by isolate source.**

Percentages of isolates susceptible to ampicillin-sulbactam (SAM-S) or amoxicillin-clavulanate (AMC-S) are listed along with absolute numbers of isolates over total numbers of isolates in parentheses. Significance of differences between percent susceptibility to SAM and AMC was determined by the McNemar test with Yates’s continuity correction. P-values were adjusted for multiple comparisons using the Bonferroni correction. For each comparison, significance is indicated after the AMC susceptibility percentage: *, p < 0.05; ***, p < 0.001; superscript ns, not significant (p > 0.05); superscript N/A, McNemar test invalid because there were no isolates with discordant SAM and AMC results. ICU, intensive care unit.

|  |  | Source | | | | |
| --- | --- | --- | --- | --- | --- | --- |
|  |  | Blood | Respiratory | Urine | Wound, tissue, body fluid | Other |
| *Citrobacter amalonaticus*  complex | SAM-S | 33%  (1/3) | 50%  (1/2) | 55%  (33/60) | 63%  (12/19) | -  (0 isolates) |
|  | AMC-S | 33%^N/A^  (1/3) | 50%^N/A^  (1/2) | 63%^ns^  (38/60) | 74%^ns^  (14/19) | -  (0 isolates) |
| *Citrobacter*  *koseri* | SAM-S | 100%  (15/15) | 93%  (55/59) | 97%  (439/453) | 97%  (110/113) | 100%  (5/5) |
|  | AMC-S | 100%^N/A^  (15/15) | 93%^ns^  (55/59) | 99%^ns^  (449/453) | 96%^ns^  (109/113) | 100%^N/A^  (5/5) |
| *Escherichia*  *coli* | SAM-S | 49%  (518/1,049) | 41%  (114/280) | 59%  (12,223/20,701) | 53%  (708/1,333) | 64%  (72/113) |
|  | AMC-S | 80%***  (840/1,049) | 68%***  (189/280) | 86%***  (17,858/20,701) | 81%***  (1,074/1,333) | 84%***  (95/113) |
| *Klebsiella oxytoca* | SAM-S | 62%  (41/66) | 71%  (84/119) | 68%  (347/509) | 75%  (141/187) | 67%  (6/9) |
|  | AMC-S | 83%*  (55/66) | 90%***  (107/119) | 91%***  (462/509) | 94%***  (175/187) | 89%^ns^  (8/9) |
| *Klebsiella pneumoniae*  group | SAM-S | 76%  (440/581) | 69%  (339/489) | 78%  (3,778/4,858) | 75%  (491/656) | 78%  (36/46) |
|  | AMC-S | 88%***  (510/581) | 83%***  (405/489) | 90%***  (4,366/4,858) | 85%***  (557/656) | 89%^ns^  (41/46) |
| *Proteus*  *mirabilis* | SAM-S | 87%  (87/100) | 86%  (89/103) | 88%  (2,103/2,392) | 89%  (504/568) | 95%  (21/22) |
|  | AMC-S | 93%^ns^  (93/100) | 93%^ns^  (96/103) | 96%***  (2,288/2,392) | 95%***  (538/568) | 100%^ns^  (22/22) |
| *Proteus*  *vulgaris* | SAM-S | 83%  (10/12) | 0%  (0/2) | 86%  (90/105) | 73%  (44/60) | -  (0 isolates) |
|  | AMC-S | 92%^ns^  (11/12) | 100%^ns^  (2/2) | 90%^ns^  (94/105) | 83%^ns^  (50/60) | -  (0 isolates) |
